# Identification of Genetic Biomarkers for Diagnosis of Myocardial Infarction Compared with Angina Patients

**DOI:** 10.1155/2020/8535314

**Published:** 2020-11-10

**Authors:** Yuanyuan Zhang, Chunyang Tian, Xuejian Liu, He Zhang

**Affiliations:** ^1^Department of Cardiology, Shengjing Hospital of China Medical University, Shenyang, Liaoning, China; ^2^Department of Pulmonary and Critical Care Medicine, Shengjing Hospital of China Medical University, Shenyang, Liaoning, China

## Abstract

**Background:**

Myocardial infarction (MI) is the most terrible appearance of cardiovascular disease. The incidence of heart failure, one of the complications of MI, has increased in the past few decades. Therefore, the identification of MI from angina patients and the determination of new diagnoses and therapies of MI are increasingly important. The present study was aimed at identifying differentially expressed genes and miRNAs as biomarkers for the clinical and prognosis factors of MI compared with angina using microarray data analysis.

**Methods:**

Differentially expressed miRNAs and genes were manifested by GEO2R. The biological function of differentially expressed genes (DEGs) was examined by GO and KEGG. The construction of a protein-protein network was explored by STRING. cytoHubba was utilized to screen hub genes. Analysis of miRNA-gene pairs was executed by the miRWalk 3.0 database. The miRNA-target pairs overlapped with hub genes were seen as key genes. Logistic regressive analysis was performed by SPSS.

**Results:**

A number of 779 DEGs were recorded. The biological function containing extracellular components, signaling pathways, and cell adhesion was enriched. Twenty-four hub genes and three differentially expressed miRNAs were noted. Eight key genes were demonstrated, and 6 out of these 8 key genes were significantly related to clinical and prognosis factors following MI.

**Conclusions:**

CALCA, CDK6, MDM2, NRXN1, SOCS3, VEGFA, SMAD4, NCAM1, and hsa-miR-127-5p were thought to be potential diagnosis biomarkers for MI. Meanwhile, CALCA, CDK6, NRXN1, SMAD4, SOCS3, and NCAM1 were further identified to be potential diagnosis and therapy targets for MI.

## 1. Introduction

Cardiovascular disease is one of the severe diseases and a capital reason of morbidity and fatality worldwide. Angina as well as MI is traditionally due to the presence of flow-limiting epicardium coronary artery stenosis [1, 2]. However, MI is the most frightful appearance of cardiovascular disease. On most occasions, MI is due to the rupture of a fragile atherosclerotic plaque; however, the plaque in angina patients tends to have substantial fibrous caps that are less possible to crack [3]. In the clinic, the incident of MI is frequently unanticipated and sudden. The diagnostic and therapeutic guidelines for both US and Europe advocate that reperfusion treatment including coronary angiography and/or percutaneous coronary intervention be administered within 2 to 24 hours for MI [4]. Even though momentous therapeutic regimens are proceeding in decade years, morbidity and mortality of MI remain high. Thus, the development of strategies for the identification of MI from angina patients to prevent the occurrence of sudden mortality is increasingly imperative.

Clinically, cardiac troponins and creatine kinases have been identified as the most mainstream diagnosis of MI [5]. However, their sensitivity and specificity may be limited, as increased cardiac troponin levels are also noted in patients with nonacute coronary syndrome, such as severe infection, renal failure, and congestive heart failure [6, 7]. Genetic factor is one of the strong cardiovascular risk factors for MI [8]; consequently, the identification of new genetic biomarkers in the diagnosis of MI remains needed.

Today, there is a growing interest in recognizing gene expression profiles mainly via microarray analysis for the identification of MI. Colpaert and Calore found that miR-133 and miR-499 were frequently associated with MI [9]. Wu et al. found that 3 genes were promising ponderable diagnostic targets of MI opposed to healthy controls [10]. However, the control groups of these studies were mostly healthy people, and few studies on clinical prognostic indicators were related to MI.

Our study made an analysis of microarray data of MI compared with angina patients with the aim of identifying differentially expressed genes and miRNAs as biomarkers for the clinical and prognosis factors.

## 2. Materials and Methods

### 2.1. Identification of DEGs

In the present study, we sifted datasets from the publicly available Gene Expression Omnibus (GEO) database (http://www.ncbi.nlm.nih.gov/geo/). First, we searched the GEO database, entering the search term “acute myocardial infarction”; filters of publication date were set to be before April 22, 2019; and the organisms were characterized as “homo sapiens.” By selecting “series,” a total of 53 series were screened out. Among them, 27 were not related to myocardial infarction, 19 were nonangina pectoris in the control group, 1 was a nonblood specimen, 1 was noncomplete gene sequencing, and 1 was duplicate data, and finally, 4 series were left: GSE60993, GSE62646, GSE29111, and GSE53211. Then, all selected datasets were peripheral blood samples from the MI group comparing with the angina group without a history of MI. Due to the large number of sample groups in the dataset, we selected the grouping to be relatively simple to analyze the ideal difference genes, so we finally chose the following two independent datasets which were screened: the mRNA dataset GSE29111 contained 36 MI patients and 16 angina samples and the miRNA dataset GSE53211 contained 9 MI patients and 5 angina samples. And GEO2R was used for screening DEGs out between MI patients and angina samples in GSE29111 with *P* value < 0.05 and ∣log(foldchange) | >1. In datasets GSE29111 and GSE53211, samples were acquired on the platforms Affymetrix Human Genome U133 Plus 2.0 Array and Exiqon Human PCR panels I+II, V1.R (miRBase version 12), respectively.

### 2.2. Functional Annotation and Pathway Enrichment of DEGs

To obtain the underlying biological function and signaling pathways of DEGs, the Database for Annotation, Visualization, and Integrated Discovery (DAVID [11], version6.8, https://david.ncifcrf.gov/home.jsp) was used for Gene Ontology (GO) annotation and Kyoto Encyclopedia of Genes and Genomes (KEGG) pathway enrichment analysis [12]. In our study, we uploaded 866 DEGs in the gene list > select identifier-official-gene-symbol > list type-gene list > submit list > species-Homo sapiens > functional annotation chart > export and save results. Only those terms or pathways with *P* value < 0.05 were selected as substantially enriched.

### 2.3. Protein-Protein Interaction Network Construction

Search Tool for the Retrieval of Interacting Genes (STRING) database [13] was used to retrieve the predicted interactions between proteins encoded by all screened DEGs and other proteins. The PPI network was visualized by the Cytoscape software (version 3.7.1). Subsequently, the plugin cytoHubba [14] of the Cytoscape software was used to select important hub genes among these genes. The hub genes were selected with the criteria of top 50 genes according to 6 cytoHubba ranking algorithms including degree, EPC (Edge Percolated Component), closeness, radiality, betweenness, and stress.

### 2.4. Differentially Expressed miRNAs

The distinction of differentially expressed miRNAs of MI patients compared with angina samples in GSE53211 was screened out by using the online tool GEO2R. The threshold was set as ∣log(foldchange) | >2 and *P* value < 0.05.

### 2.5. Hub Genes Regulated by miRNA

Herein, the miRNA-target prediction tool miRWalk 3.0 [15] database was implemented to forecast the target genes of differentially expressed miRNAs. Forecasting target genes that overlapped with hub genes were regarded as key genes. Then, differentially expressed miRNAs and the hub gene interaction network were constructed and visualized by Cytoscape 3.7.1.

### 2.6. The Logistic Regression of Key Gene Expressions and Clinical and Prognosis Factors

All statistical analyses were conducted and processed using the SPSS statistical software version 17.0. The association between key gene expression and clinical and prognosis factors including heart failure, BMI (Body Mass Index) value, CRP (C-Reactive Protein) value on admission, and EF (ejection fraction) value was evaluated with binary logistic regression to screen independent predictive indicators. All covariates were set as categorical data. Statistical significance was identified at *P* ≤ 0.05, and *α* = 0.05 was taken as the significant level.

Greater than the average expression level of key genes was determined as overexpression, while less than the average expression level was regarded as low expression. The BMI and CRP (mg/l) were considered a high value if their laboratory level was greater than 28 [16] and 2.8, respectively. EF value less than 50% was considered heart failure [17]. The World Health Organization (WHO) has proposed BMI = 30kg/m^2^ as the critical value of obesity for adults in Western countries [16]. However, according to the “Chinese Expert Consensus of Overweight/Obesity Medical Nutritional Therapy, 2016 Edition,” among people aged 18-70, obesity is defined as BMI ≥ 28kg/m^2^. Therefore, we choose a BMI of 28. And the median CRP value in the original data was 2.83. Our analysis found that regardless of the value of 2.8 or 2.83, their conclusions are the same, so we selected 2.8 after rounding as the standard. According to the practice guidelines of the American College of Cardiology/American Heart Association and European Heart Association, heart failure is defined by clinical heart failure syndrome with LVEF < 50% [17]. Therefore, we choose 50% as the critical value of the EF indicator.

## 3. Results

### 3.1. Identification of DEGs

**Figure 1:**
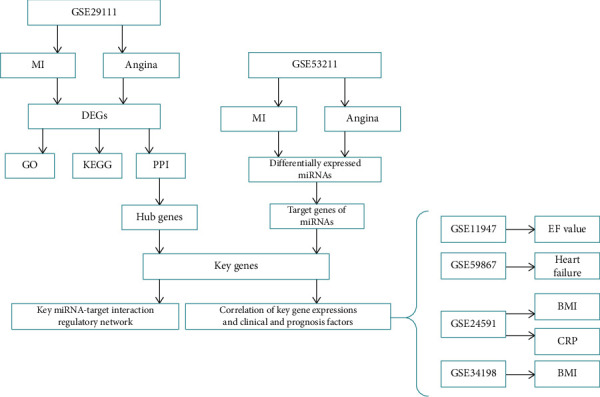
The bioinformatics analysis flowchart.

The bioinformatics analysis flowchart was carried out as shown in Figure 1. Taking advantage of the microarray dataset GSE29111, consequently, a number of 779 DEGs were identified using GEO2R. GEO2R, an interactive tool for the GEO database, was used for screening DEGs out between MI patients and angina samples in GSE29111 with *P* value < 0.05 and ∣log(foldchange) | >1. Among them, we found 393 genes with upregulation and 386 genes with downregulation. The top 10 DEGs with up- or downregulation are shown in Table 1.

### 3.2. Functional Annotation and Pathway Enrichment of DEGs

**Figure 2:**
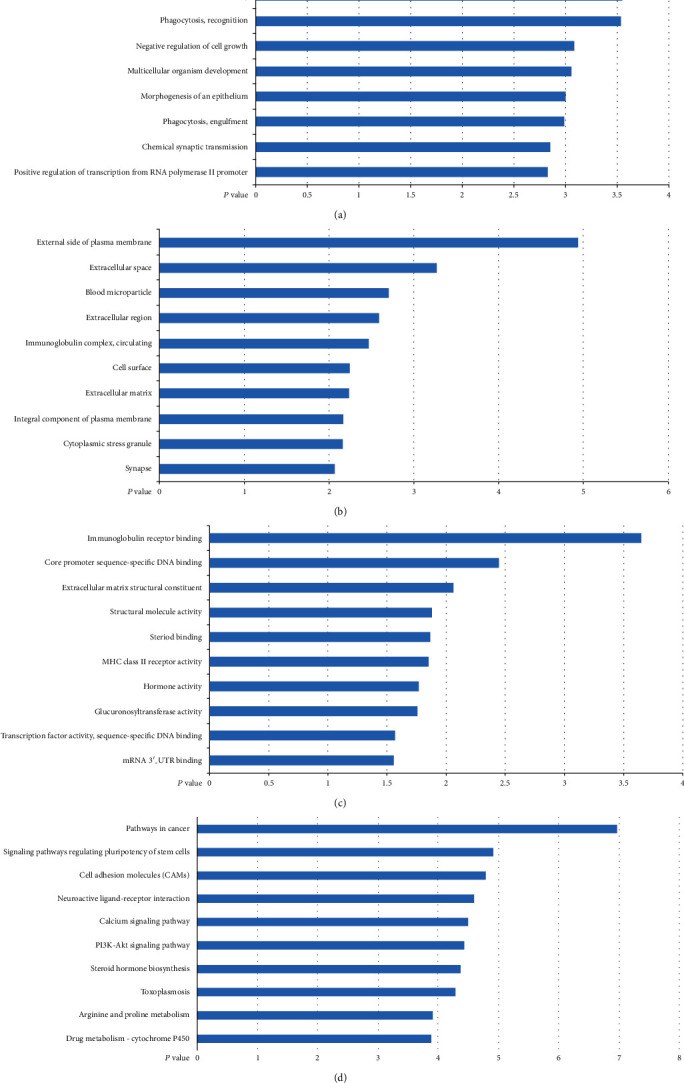
Top 10 significant enrichment GO and KEGG terms of DEGs in GSE29111: (a) BP: biological process; (b) CC: cellular component; (c) MF: molecular function; (d) KEGG: signaling pathway. The vertical axis represents the GO or KEGG pathway terms; the horizontal axis shows the negative log10 (*P* value).

To explore the possibility biological function of the DEGs, GO analysis was applied by DAVID. A *P* value of <0.05 was recognized as significant. The top ten enriched GO terms consisted of biological process (BP), cellular component (CC), and molecular function (MF) ontology which are demonstrated in Figures 2(a)–2(c).

The result revealed that CC associated with extracellular components containing extracellular space, extracellular region, and extracellular matrix were significantly enriched. In addition, BP items related to phagocytosis and cell growth, for instance, phagocytosis recognition, phagocytosis engulfment, negative regulation of cell growth, and multicellular organism development, were obviously screened out. Furthermore, the main constitution in MF items was binding-related ontologies, such as immunoglobulin receptor binding, core promoter sequence-specific DNA binding, steroid binding, and mRNA 3′-UTR binding.

To understand the more advanced pathway information of the DEGs, KEGG pathway analysis was performed by using DAVID. Functional classification of these 779 DEGs indicated significant enrichment in 10 KEGG pathways (Figure 2(d)). It can be observed that the majority of enriched KEGG functional categories, including signaling pathways regulating pluripotency of stem cells and cell adhesion molecules (CAMs), were found to be associated with signaling pathways and cell adhesion involved in MI.

### 3.3. Protein-Protein Interaction Network Construction

**Figure 3:**
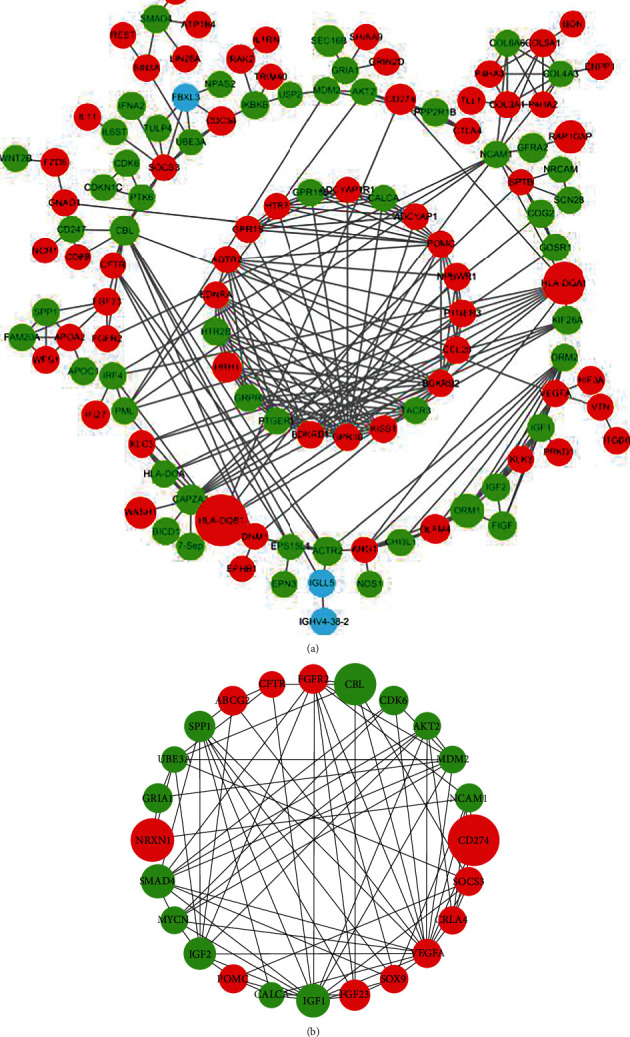
The PPI network of DEGs and top 24 hub genes: (a) the PPI network of DEGs; (b) the PPI network of top 24 hub genes. (The size of each node is proportional to the |log(fold change)| value. The green color indicates downregulation, and the red color indicates upregulation. The blue color indicates non-DEGs).

To acquire the communication between the DEG-encoded proteins in GSE29111 and additional proteins, the PPI network was investigated and visualized by Cytoscape.  Figure 3(a) shows this PPI network with the combined score no less than 0.9, which consisted of 121 nodes and 276 edges.

Furthermore, cytoHubba analyzed the PPI network and ranked all nodes according to six scoring algorithms. At last, twenty-four genes with higher scores were screened out.  Figure 3(b) shows the PPI network of the top 24 genes established by Cytoscape. All of these twenty-four top related genes were DEGs. Eleven out of these twenty-four genes were overexpressed and thirteen were low expressed.

### 3.4. Differentially Expressed miRNAs

To screen the differentially expressed miRNAs of MI compared with angina, the miRNA profiling dataset GSE53211 was analyzed using the GEO2R tool. As a consequence, we observed a total of 3 differentially expressed miRNAs and they were entirely upregulated in MI samples compared with angina samples (Table 2).

### 3.5. Hub Genes Regulated by miRNA

**Figure 4:**
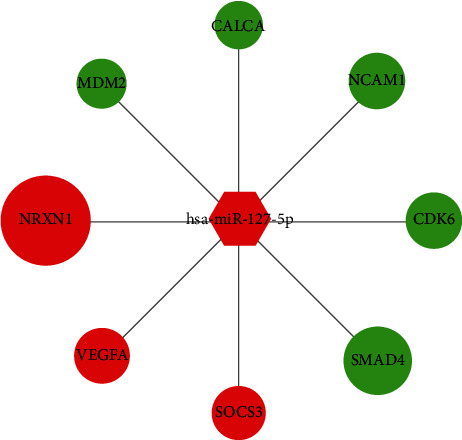
The key miRNA-target interaction regulatory network. (The size of each node was proportional to |log(fold change)| value. The green color indicates downregulation, and the red color indicates upregulation. The circular node represents key mRNA, and the hexagon node represents miRNA.)

To further understand the regulatory relationship between hub genes and differentially expressed miRNAs, by searching these 3 miRNAs in the miRWalk 3.0 database, a total of 3786 genes were observed. Moreover, nine miRNA-target pairs were subsequently overlapped with the 24 hub genes mentioned above, and the overlapped 8 hub genes were considered key genes (Table 3). Interestingly, they were all regulated by hsa-miR-127-5p. Three out of these eight key genes were upregulated, and the other five were downregulated. Additionally, the regulatory network of key miRNA-target interaction is presented in Figure 4.

### 3.6. The Logistic Regression of Key Gene Expressions and Clinical and Prognosis Factors

To determine the risk factors between key gene expressions and clinical and prognosis factors in MI, 4 datasets including GSE24591, GSE59867, GSE34198, and GSE11947 were performed by logistic regressive analysis.

As shown in Table 4, MI patients with an EF value greater than 50 showed lower expression of NCAM1 than patients with an EF value less than 50. The probability of heart failure was significantly increased in patients with high expression of SOCS3 and NRXN1, while the expression of CDK6 and SMAD4 was significantly reduced in patients without heart failure. High CRP value patients tended to express low levels of SMAD4 and distinguished levels of NCAM1 compared with low CRP value patients. In addition, BMI value was positively associated with CALCA status in GSE34198 and negatively associated with NCAM1 status in GSE24591. It is worth noting that NCAM1 was found to have significant correlation with 3 clinical laboratory and prognosis factors, including BMI, CRP on admission, and EF value. Combined with this information, logistic regression identified NCAM1 as potentially suitable therapeutic targets for MI patients.

## 4. Discussion

Barnett provided the latest systematic review of acute myocardial infarction [3]. MI may unpredictably induce heart failure, sinus arrest, arrhythmia, or cardiogenic shock. As a disease with a high incidence rate of serious hemodynamic instability and sudden death, even though momentous therapeutic regimens including urgent PCI surgery within 24 hours are proceeding in decade years, MI is one of the principal reasons of death all over the world. Clinically, cardiac troponins and creatine kinases have been identified as the most mainstream diagnosis of MI. However, their sensitivity and specificity may be restricted. Today, there is a growing interest in recognizing gene expression profiles mainly via microarray analysis for the identification of MI compared with healthy people. However, there are few genetic studies comparing MI with angina pectoris. Therefore, it is necessary to observe new biomarkers by microarrays, which will enhance the development of strategies for MI.

In the present study, we obtained 2 datasets from GEO by repurposing microarray data and compared gene expression profiles between MI and angina samples. We concluded that 8 key genes and 1 key miRNA are differentially expressed in MI compared with angina samples. Depending on the functional enrichment analysis, we observed that key genes were functionally enriched for hormone activity, cell surface, and PI3K-Akt signaling pathway. Finally, we identified the correlativity between key gene expressions and clinical and prognosis factors, including the EF value, heart failure, CRP value, and BMI value by logistic regressive analysis.

CDK6 encodes an enzyme regulated by cyclins, which are known to be important regulators of cell cycle progression. It was pointed out that upregulation of CDK6 and activation of the PI3K/AKT pathway alleviated hypoxia-induced pheochromocytoma cell injury [18]. The downregulation of CDK6 was necessary to demonstrate remarkably deteriorating cardiovascular disease risk by destroying the vascular endothelial structure and function [19]. Moreover, the replicating biological process of fibroblasts in mouse embryo was intensified possibly by containing CDK6 expression [20]. In our study, the expression of CDK6 decreased significantly likely because of the enrichment of the PI3K/AKT pathway at an early stage of MI. Likewise, in the status of heart failure during MI convalescence, the expressions gradually decreased probably due to ventricular remodeling.

The protein encoded by SOCS3 is a cytokine-inducible negative regulator of cytokine signaling. This protein can attach to the JAK2 kinase and curb the capability of the JAK2 kinase. It was found that the protection of the myocardium in opposition to the left ventricle damage induced by MI was employed though stimulation of JAK2/STAT3 signaling and coexisted with low expression of SOCS3 [21]. Additional studies suggested that SOCS3 gene silencing in rats with heart failure greatly decreased myocardial fibrosis as well as the inflammatory response, leading to advancement of heart function [22]. This outcome was supported by the current study, which demonstrated that SOCS3 was simultaneously upregulated in MI and heart failure patients. There was the possibility that SOCS3 dominated the pathological angiogenesis by inhibition of JAK/STAT signals [23], sequentially repaired cardiac remodeling, and inflammation.

NCAM1 encodes a cell adhesion protein located on the cell surface and plays a major role in cell-cell adhesion, neurite outgrowth, synaptic plasticity, and learning and memory. Previous studies showed that NCAM1 levels were lower in coronary artery disease and significantly related to a high hazard of coronary artery disease [24]. The NCAM1-negative NK cells were observed to be increased in patients with MI, which were associated with higher creatine kinase levels, suggesting plaque instability, thrombi, and myocardial damage [25]. Additional studies suggested that monomeric CRP in mouse extracellular matrix increased NCAM expression, associating with microvasculature plaques [26]. NCAM1-positive circulating cell phenotypes in the monocyte and neutrophil cells appeared to be associated with significantly increased levels of CRP, suggesting a circulating immunocompromised state and systemic inflammation [27]. Moreover, NCAM1 demonstrated significant overexpression in weight loss [28]. In line with the above study, the present study demonstrated that NCAM1 was low expressed and markedly enriched in the cell surface in MI samples. Similarly, this work revealed a causal relationship between increased NCAM1 levels and low BMI value, which oppositely occurred with the CRP value in MI patients. Moreover, we observed that NCAM1 was radically low expressed in patients suffering heart failure. The precise mechanism is unclear but probably involves the inflammatory response activation at the time of MI and inhibition in the ensuing post-MI remodeling phase, as well as the immune system under both homeostatic and perturbed conditions, requiring further study.

SMAD4 encodes a protein implicated in cell signaling in mammals. It is owned by the proteins of the SMAD family. The proteins encoded by SMAD4 have a definite effect in reducing angiogenesis and increasing blood vessel hyperpermeability. It was found that the SMAD pathway was an independent regulatory pathway exerting on the development process of coronary vessels, which contributed to vessel growth after MI in infants [29]. Cardiovascular abnormalities of Myhre syndrome were related to the capability of the SMAD4 encoded protein to harmonize various signaling pathways. And the gene product of SMAD4 was critical to cardiovascular homeostasis [30]. Furthermore, SMAD4 knock-off mice had a significant decline in the left ventricular ejection fraction, indicating that SMAD4 was critical for maintaining cardiac function and cardiomyocyte survival [31]. Previous studies also showed that the aggression of arteriosclerosis was due to the sustainment of inflammatory monocytes and the disruption of monocyte homeostasis through the reduction of SMAD4 levels [32]. Our results showed the same result in that the downregulated gene SMAD4 in MI samples was implicated in heart failure and low CRP value. However, SMAD4 and NCAM1 were paradoxically expressed in the CRP level, although they were both found to be potential genetic biomarkers of MI protection. In fact, the precise role inflammation plays in the setting of MI has been debated since the 1980s with the inflammatory cells being recognized as a double-faced role of MI, notably, their ability to induce either proinflammatory or reparative phase of cardiomyocytes. However, there is an increasing amount of experimental evidence that a high CRP level in peripheral blood after MI predicted an enhanced inflammatory response, which was associated with a poor clinical outcome, for example, cardiac rupture, ventricular aneurysm constitution, and deterioration of left ventricular rebuilding [33].

MDM2 encodes a nuclear-localized E3 ubiquitin ligase. It was reported that apoptosis rates of myocardial cells increased in heart failure rats, through inhibition of the expression of MDM2, which was the inhibitor of P53 [34]. In addition, ATP depletion in human umbilical vein endothelial cells (HUVEC) inhibited the PI3K/Akt pathway and MDM2 activity, sequentially inducing endothelial apoptosis [35]. Niu et al. also found that the stimulation of the PI3K/Akt pathway mediated HUVEC migration and improved wound healing [36]. Herein, we also found that MDM2 was downregulated and significantly enriched in the PI3K/Akt signaling pathway in MI samples.

VEGFA is a part of the platelet-derived growth factor/vascular endothelial growth factor (PDGF/VEGF) family. PDGF/VEGF motivates migration and multiplication of vascular endothelial cells and is necessary for physiological as well as pathological angiogenesis. It was necessary to demonstrate that VEGFA noticeably inhibited the human macrophage migration in myocardial tissue after MI through restraint of the ERK/NF-*κ*B signaling pathways [37]. Macrophages were reported to promote angiogenesis, vascular permeability, and inflammatory cell recruitment via the activation of VEGFA, inducing human atherosclerotic plaque progression and rupture [38]. Furthermore, the oxidative stress induced vascular permeability factor VEGFA secretion, which was related to increased myocardial infarctions [39]. In our study, we observed that VEGFA was upregulated in MI patients, which was also supported by the above studies.

CALCA encodes calcitonin, which is a polypeptide hormone. de Boer et al. performed a prospective study of heart failure patients presenting with respiratory symptoms. CALCA-guided treatment resulted in improved outcomes, including admission to the intensive care unit and mortality at 30 days [40]. The overexpression of CALCA was explored to be correlated with a high left ventricular ejection fraction value, which predicted better cardiac function and less severity of illness [41]. In addition, the increased serum CALCA levels were extremely related to obesity [42], which was supported by the present study. It has been argued that obesity is a risk factor of MI [43] and consequent ischemic heart disease [44], but those findings remain controversial. Indeed, it has been argued that low BMI is associated with mortality from coronary disease [45]. In the present study, we also found that CALCA was low expressed in MI patients and functionally enriched in hormone activity. This might be attributed to cardioprotective effects, including decreasing oxidative stress and reducing reactive oxygen species production.

The protein encoded by NRXN1 acts on the vertebrate nervous system as cell adhesion molecules and receptors. NRXN1 was reportedly associated with neurological diseases, such as tardive dyskinesia and schizophrenia; however, there were no reports about NRXN1 in the field of coronary heart disease; besides, it was reported that NRXN1 was associated with high nicotine addiction [46]. As we all know, smoking represents an independent risk factor resulting in cardiovascular disease. Depending on our results, the level of NRXN1 was concurrently found to be overexpressed in MI and heart failure samples. Read in conjunction, NRXN1 may induce cardiovascular diseases through some mechanism, and further exploration is needed to lucubrate the cardiovascular function of NRXN1.

miR-127-5p regulated all these eight key genes in the present study. It is noteworthy that miR-127 [47], VEGFA [37], and SOCS3 [48] all impacted on the NF-*κ*B pathway. Overall, we deduced under the condition of coronary artery anoxia that miR-127 not only enhanced VEGFA secretion and SOCS3 promoter methylation but also inhibited the expression of SOCS3 through the JAK/STAT3/NF-*κ*B pathway, leading to pathological angiogenesis, which was involved in left ventricular injury, coronary artery hyperplasia, and restenosis after MI, consistent with the research conclusion of Boosani et al. [48]. Some limitations of our study should be acknowledged. Although the above result based on bioinformatics analysis is important, confirmation of the above-mentioned results is required by performing functional studies, and the specific and precise possible mechanism of these potential biomarkers requires our follow-up experiments to verify. Additional clinical studies are also required to confirm the above findings.

## 5. Conclusions

In conclusion, we made a detailed analysis of these datasets through bioinformatics, and the latent function of these recognized differentially expressed genes and miRNA would be validated by animal and cell culture models in our future work. Overall, CALCA, CDK6, MDM2, NRXN1, SOCS3, VEGFA, SMAD4, NCAM1, and miR-127-5p were considered to be potential genetic diagnosis biomarkers for MI, which could help to distinguish MI from angina patients from the perspective of genetics and improve accurate diagnosis and therapies of MI.

## Figures and Tables

**Table 1:** Top 10 up- and downregulated DEGs in GSE29111.

Gene symbol	*P* value	LogFC	Gene function	PubMed IDs
Upregulated				
TSTA3	1.29*E*-05	1.0854319	GDP-4-dehydro-D-rhamnose reductase activity	11698403
SLC22A11	2.64*E*-05	1.7937471	Organic anion transmembrane transporter activity	15102942
ALDH5A1	3.94*E*-05	1.1577679	Protein homodimerization activity	16199352
PLA2G16	5.57*E*-05	1.2325045	Catalyzes the release of fatty acids from phospholipids in adipose tissue	17374643
DTNA	7.35*E*-05	2.0915867	Clustering of nicotinic acetylcholine receptors	11353857
HIF3A	7.63*E*-05	1.4873978	Adaptive response to hypoxia	11573933
POLL	1.25*E*-04	1.3674519	Repair polymerase	10982892
CTDSPL	1.42*E*-04	2.1596408	Negatively regulates RNA polymerase II transcription	20379614
ENDOV	1.43*E*-04	1.6507937	Cleaves inosine-containing RNAs	23912683
THSD4	1.49*E*-04	2.1230904	Promotes FBN1 matrix assembly	19734141
Downregulated				
KYAT1	8.00*E*-06	-2.1991848	CCBL activity and transaminase activity with many amino acids	19826765
LOC101927044	1.48*E*-05	-1.4311556	Uncharacterized	—
ADAMTS9	1.58*E*-05	-1.5421577	Metallopeptidase activity	12514189
ETS1	2.41*E*-05	-1.1693245	Transcription factor binding	10698492
PNMA3	2.82*E*-05	-1.4922227	Nucleic acid binding	16344560
MARC2	4.20*E*-05	-2.2687039	Nitrate reductase activity	20808825
HSD17B12	4.54*E*-05	-2.1426028	Long fatty acid elongation	20937905
SCRT1	4.62*E*-05	-1.489692	Transcriptional repressor	11274425
PNMA6A	6.01*E*-05	-1.3616952	Functional ribosomal frameshift signal	16407312
APOC1	8.06*E*-05	-1.16521	Fatty acid binding	17339654

**Table 2:** Differentially expressed miRNAs in GSE53211.

miRNA	*P* value	LogFC
hsa-miR-337-5p	0.000244	2.24308
hsa-miR-127-5p	0.001786	2.3002
hsa-miR-539	0.048052	2.23645

**Table 3:** Hub genes in GSE29111 regulated by hsa-miR-127-5p in GSE53211.

ID	*P* value	*t*	*B*	LogFC	Gene symbol	Gene title	Gene function
210727_at	0.0137	2.5508641	-2.99651	-1.0294165	CALCA	Calcitonin-related polypeptide alpha	Dilates vessels including the coronary, cerebral, and systemic
235287_at	0.00177	3.297353	-1.26724	-1.1501461	CDK6	Cyclin-dependent kinase 6	Cell cycle and differentiation; delays senescence
205386_s_at	0.01	2.6727568	-2.73565	-1.042301	MDM2	MDM2 protooncogene	Mediates ubiquitination and subsequent proteasome degradation of DYRK2 in nucleus
1558708_at	0.00553	-2.8958494	-2.23542	1.6471658	NRXN1	Neurexin 1	Synaptic signal transmission
206359_at	0.0283	-2.2568286	-3.58693	1.1075956	SOCS3	Suppressor of cytokine signaling 3	Inhibits cytokine signal transduction by binding to tyrosine kinase receptors
211527_x_at	0.0264	-2.2854412	-3.53199	1.1348245	VEGFA	Vascular endothelial growth factor A	Induces endothelial cell proliferation, promotes cell migration, inhibits apoptosis, and induces permeabilization of blood vessels
1565702_at	0.0295	2.2381206	-3.62256	-1.3151998	SMAD4	SMAD family member 4	TGF-mediated signaling
214952_at	0.0398	2.1090612	-3.86171	-1.1379034	NCAM1	Neural cell adhesion molecule 1	Cell adhesion molecule

**Table 4:** The logistic regression of key gene expressions and clinical and prognosis factors in MI.

Dataset	Factors	Gene	*B*	S.E.	Wald	Sig.	OR	95% CI for EXP(*B*)
GSE11947	EF value	NCAM1	-1.859	0.949	3.838	0.05	0.156	0.024-1.001
GSE59867	Heart failure	SOCS3	1.026	0.518	3.928	0.048	2.79	1.011-7.698
		NRXN1	1.026	0.518	3.928	0.048	2.79	1.011-7.698
		CDK6	-1.299	0.528	6.055	0.014	0.273	0.097-0.768
		SMAD4	-1.585	0.541	8.57	0.003	0.205	0.071-0.592
GSE24591	CRP on admission	NCAM1	1.705	0.801	4.534	0.033	5.5	1.145-26.412
		SMAD4	-1.705	0.801	4.534	0.033	0.182	0.038-0.873
	BMI	NCAM1	-1.705	0.801	4.534	0.033	0.182	0.038-0.873
GSE34198	BMI	CALCA	1.542	0.707	4.759	0.029	4.675	1.17-18.686

## Data Availability

The data used to support the findings of this study can be found in https://figshare.com/s/bb087401e2fc0a7a752d.
